# The Effects of *Aronia melanocarpa* Juice Consumption on the mRNA Expression Profile in Peripheral Blood Mononuclear Cells in Subjects at Cardiovascular Risk

**DOI:** 10.3390/nu12051484

**Published:** 2020-05-20

**Authors:** Ljiljana Stojković, Ivan Jovanović, Maja Zivković, Manja Zec, Tamara Djurić, Ivan Zivotić, Jovana Kuveljić, Ana Kolaković, Ivana Kolić, Ana Djordjević, Marija Glibetić, Dragan Alavantić, Aleksandra Stanković

**Affiliations:** 1 Laboratory for Radiobiology and Molecular Genetics, “Vinča” Institute of Nuclear Sciences, University of Belgrade, 11000 Belgrade, Serbia; ljiljanas@vinca.rs (L.S.); ivanj@vinca.rs (I.J.); majaz@vinca.rs (M.Z.); tamariska@vinca.rs (T.D.); ivanz@vinca.rs (I.Z.); jovana@vinca.rs (J.K.); anakolakovic@vinca.rs (A.K.); ivanak@vinca.rs (I.K.); ana.djordjevic@vinca.rs (A.D.); adragan@vinca.rs (D.A.); 2 Centre of Research Excellence in Nutrition and Metabolism, Institute for Medical Research, University of Belgrade, 11000 Belgrade, Serbia; manjazecimr@gmail.com (M.Z.); mglibetic@gmail.com (M.G.)

**Keywords:** *Aronia melanocarpa*, polyphenol, peripheral blood mononuclear cells, messenger RNA (mRNA), transcriptome, cardiovascular risk

## Abstract

Foods and food products that contain polyphenols are proposed to modulate risk of cardiovascular disease. The aim of this three-arm, crossover, randomized, double-blind, placebo-controlled intervention study was to examine the impact of *Aronia melanocarpa* juice (AMJ), high-polyphenol (AMJ treatment, 1.17 g/100 mL polyphenols) and low-polyphenol (dAMJ treatment, 0.29 g/100 mL polyphenols) dose, on the transcriptome in peripheral blood mononuclear cells (PBMC) of 19 subjects at cardiovascular risk. Transcriptome data were obtained by microarray. Bioinformatic functional annotation analysis was performed on both the whole transcriptome datasets and the differentially expressed genes (DEGs). Expression of selected DEGs was validated by RT-qPCR. Administration of AMJ and dAMJ treatments during the two consecutive four-week treatment periods had additive effects on PBMC transcriptome profiles, with the most pronounced and specific effect noticed for AMJ in the last treatment period (TP3) of the trial. Between the high-dose and low-dose treatments in TP3, there was a multitude of overlapping DEGs and DEG-enriched biological processes and pathways, which primarily included immunomodulation and regulation of cell proliferation/death. Increased expression of *TNF*, *IL1B*, *IL8*, *RGS1*, *OSM*, and *DUSP2* in TP3 was confirmed by RT-qPCR. The results suggest the immunomodulatory effects of prolonged habitual consumption of polyphenol-rich aronia juice in individuals at cardiovascular risk.

## 1. Introduction

The majority of premature cardiovascular diseases (CVDs) can be prevented by changes in lifestyle and diet, which is of particular importance, as CVDs represent a leading cause of death worldwide [1]. Polyphenol-containing plant foods and derived products are suggested to modulate CVD risk factors [2,3,4], but the evidence is still restricted and inconsistent. The findings also indicate that higher intake of polyphenols might be more beneficial in individuals “at cardiovascular risk” than in the healthy [5]. However, the recommended doses of specific polyphenol consumption, which would result in the maximum benefit for humans, are not yet determined [6]. Only a few trials were performed to examine the impact of well-defined doses of polyphenol-rich products on human health, compared to proper placebo without polyphenols. Generally, intervention studies are necessary to validate the biological effects of plant foods and derived products that contain polyphenols in particular subpopulations [7,8,9].

The biological effects of polyphenol application on lipopolysaccharide (LPS)-induced inflammation in peripheral blood mononuclear cells (PBMC) were beneficial [10,11]. Other in vitro studies showed that treatments with polyphenols were able to increase the constitutive and/or stimulated expression of proinflammatory genes in PBMC and epidermal cells [12,13,14,15,16]. *Aronia melanocarpa* has a higher total polyphenol content relative to other polyphenol-rich fruits [17,18]. Therefore, the present three-arm, crossover, randomized, double-blind, placebo-controlled intervention study was designed to compare the biological effects of two doses of a commercial *Aronia melanocarpa* juice. We aimed to explore the impact of a high- and a low-polyphenol dose of aronia juice (including nutritionally matched placebo beverage without polyphenols, as a control treatment) and the additive effects of the two polyphenol doses on the whole transcriptome in PBMC in individuals at risk of CVD.

## 2. Materials and Methods

### 2.1. Study Design and Intervention Treatments

**Figure 1:**
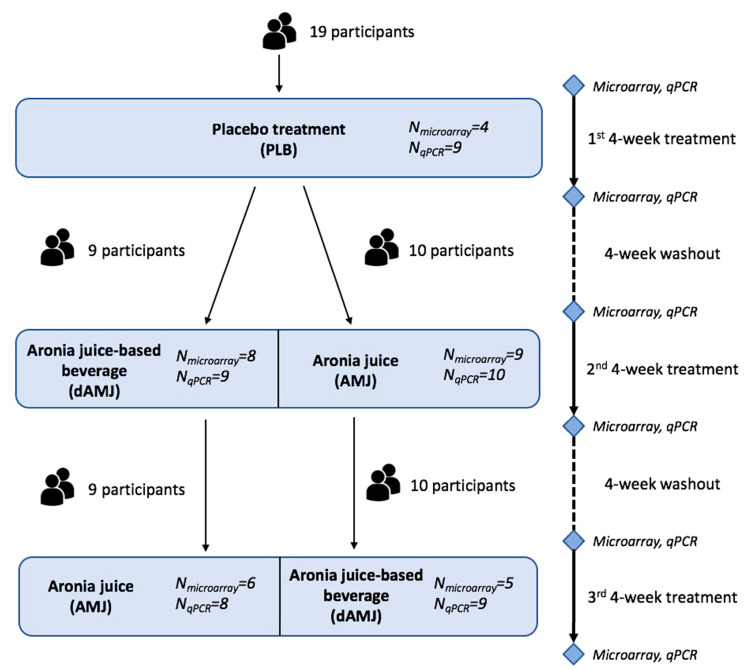
Study workflow. AMJ—treatment with original *Aronia melanocarpa* juice containing a total polyphenol amount of 11,771.09 mg gallic acid equivalent (GAE)/L, which corresponds to 1.17 g of total polyphenols per 100 mL of the allocated treatment (high dose); PLB—treatment with placebo beverage, a formulation that has the same appearance, taste, and nutritional composition of the original aronia juice, but without bioactive polyphenols; dAMJ—treatment with aronia juice-based beverage, made by diluting the AMJ with the PLB (ratio 1:3) and containing a total polyphenol amount of 2942.77 mg GAE/L, which corresponds to 0.29 g of total polyphenols per 100 mL of the allocated treatment (low dose).

The present research (Figure 1) is designed as a sub-study of the original three-arm, crossover, randomized, double-blind, placebo-controlled clinical trial registered at ClinicalTrials.gov as NCT02800967. The original study included non-smoking adults at moderate risk of CVD, defined as the presence of at least one of the following: increased body mass index (BMI) (25–30 kg/m^2^), central obesity (waist circumference ≥80 cm for women and ≥94 cm for men), and high normal blood pressure (systolic/diastolic blood pressure >120/80, ≤139/89 mm Hg). Exclusion criteria were as follows: presence of chronic or acute disease, self-reported allergy to polyphenol-rich food, pregnancy, lactation, blood donation 16 weeks before the start of the study, and parallel participation in another clinical trial. The participants were asked to follow their habitual diet and usual physical activity, but to strictly refrain from berries and berry products, during the course of the study. They were also requested to avoid excess amounts of polyphenol-rich food, inclusive of green tea, olive oil, and excessive quantities of nuts. They consumed study treatments as part of their habitual diet.

The original *Aronia melanocarpa* juice used in the study was registered at the Serbian Ministry of Health as a dietary supplement and was donated from “Nutrika” LTD (Belgrade, Serbia). During the three treatment periods of the NCT02800967 study, recruited participants were randomly assigned to the following three 100 mL/day interventions: (1) original *Aronia melanocarpa* juice (assigned as AMJ treatment), containing total polyphenol amount of 11,771.09 mg gallic acid equivalent (GAE)/L, which corresponds to 1.17 g of total polyphenols per 100 mL of the allocated treatment (high dose); (2) placebo beverage (assigned as PLB treatment), a formulation that has the same appearance, taste, and nutritional composition of the original aronia juice, but without bioactive polyphenols [19]; (3) aronia juice-based beverage (assigned as dAMJ treatment), produced by diluting the AMJ with the placebo beverage (ratio 1:3) to contain a total polyphenol amount of 2942.77 mg GAE/L, which corresponds to 0.29 g of total polyphenols per 100 mL of the allocated treatment (low dose). It was previously reported that the daily amount of 100 mL of the placebo beverage is safe for human consumption [19]. Total polyphenols in the original aronia juice were determined using a modified Folin–Ciocalteu method [20]. The study compliance was assessed according to returned empty bottles of intervention drinks and self-reports.

The study protocol adhered to the regulations of the 1975 Declaration of Helsinki, and was approved by Clinical Hospital Centre Zemun, Belgrade, Serbia, Ethics Committee Approval, No: 2125, 2013. Written informed consent was given by all participants before the commencement of the study. 

### 2.2. Sample Collection

Study participants were instructed for overnight fasting and venous blood was collected the next morning between 8:00 and 9:00 a.m., into the sample tubes for serum and ethylenediaminetetraacetic acid evacuated tubes. The sample collection was done at two time points—before and after the corresponding four-week treatments (AMJ, dAMJ, and PLB).

### 2.3. Assessment of Study Variables

For determination of baseline dietary intake, trained staff conducted structured interviews with study subjects and collected data by using a food frequency questionnaire and repeated 24-hour dietary recalls. The subjects were assisted with a 125-item photo-booklet comprising simple foods and composite dishes [21]. Data from dietary recalls were analyzed by use of the nutritional platform for comprehensive diet evaluation [21,22]. Of relevance for this study, no outliers were identified with regard to habitual dietary intakes of total energy and main nutrients, among the study subjects upon enrollment.

Bio-impedance analyzer TANITA UM072 balance (TANITA Health Equipment H.K. Ltd) was used for determination of body weight. Total cholesterol, as well as high-density (HDL-C) and low-density (LDL-C) lipoprotein cholesterols, triacylglycerols, and glucose from serum were assessed by Roche Diagnostics Kits, using the Cobas c111 analyzer (Roche Diagnostics, Basel, Switzerland). 

### 2.4. Isolation of PBMC and Preparation of the Total RNA 

The peripheral blood samples of each subject were assembled at two time points—before and after treatment, for the corresponding treatment period(s). The PBMC were isolated with lymphocyte separation medium (PAA, GE Healthcare), and the total RNA was extracted from PBMC by using TRI Reagent (Ambion, Life Technologies). The quantity of total RNA was estimated by BioSpecnano spectrophotometer (Shimadzu Biotech). Bioanalyzer 2100 was employed for the assessment of total RNA quality/integrity, by deployment of the RNA 6000 Nano Kit (Agilent, Santa Clara, CA, USA). All RNA samples had an RNA integrity number (RIN) >7 and were kept at −80 °C prior to use.

### 2.5. Samples for Microarray Analysis

The microarray analysis comprised 19 subjects (12 men and seven women). There were 32 analyzed treatments in three successive treatment periods, for which the microarray was carried out at two time points (before and after each treatment): 15 AMJ treatments, 13 dAMJ treatments, and four PLB treatments (Table S1, Supplementary Materials). We investigated the effects of AMJ and dAMJ treatments on the PBMC messenger RNA (mRNA) expression profile during the second and third treatment periods (TP2 and TP3), as well as after the PLB treatment during the first treatment period (TP1) (Figure 1). The reasons for choosing this study design were three-fold: (1) the placebo treatment prior to polyphenol-containing treatments provided us with a polyphenol-lacking placebo arm, to determine the exclusive effects of polyphenol-containing treatments; (2) the inclusion of two treatments with different polyphenol content enabled a discussion on high vs. low polyphenol dose, conferring the role of the public health relevance of the intervention effects; (3) the subsequent allocation to either low- or high-polyphenol content treatment provided us with information on the additive (cumulative) effects of polyphenol consumption. Overall, during TP1, four subjects were allocated to PLB. During TP2, there were nine subjects allocated to AMJ and eight subjects allocated to dAMJ, and, further on, six subjects allocated to AMJ and five subjects allocated to dAMJ during TP3 (Table S1, Supplementary Materials). To examine the effects of two successive treatments on the PBMC mRNA expression profile, with respect to their order in TP2 and TP3, AMJ → dAMJ or dAMJ → AMJ (after the PLB treatment during TP1), respectively, there were five subjects with an AMJ → dAMJ and six subjects with a dAMJ → AMJ successive treatment transition (Table S1, Supplementary Materials).

### 2.6. Microarray PBMC mRNA Expression Profiling and Determination of Differentially Expressed Genes

The total RNA (200 ng) from PBMC was used for amplification of the poly(A) RNA, by in vitro transcription of biotin-aRNA, using a TargetAmp™- Nano Labeling Kit for Illumina® Expression BeadChip® (Epicentre, Madison, WI, USA). Biotin-aRNA purification was done with RNeasy® MinElute® Cleanup kit (Qiagen, Hilden, Germany), and the purified biotin-aRNA was quantified using a BioSpecnano spectrophotometer (Shimadzu Biotech, Kyoto, Japan). The Whole-Genome Gene Expression Direct Hybridization Assay (HumanHT-12 v.4 Expression BeadChip Kit, Illumina Inc., San Diego, CA, USA) was used for hybridization of biotin-aRNA (750 ng) to each array. The beadchips were scanned with iScan System (Illumina Inc., San Diego, CA, USA). Initial quality control of gene expression data was done using control metrics in GenomeStudio Gene Expression Module v1.9 (Illumina Inc., San Diego, CA, USA), and subsequent quality assessment was done using the Illumina QC pipeline of the ArrayAnalysis.org tool [23]. The results from GenomeStudio Gene Expression Module v1.9 were exported and further analyzed by R/Bioconductor software [24] and *limma* v3.32.0 package for linear modeling of microarray data [25,26]. The neqc function was used for pre-processing steps (background correction, quantile normalization, log2 transformation, and removing the control probes) [27]. Unexpressed and low-expression probes were filtered out (filtering conditions: probes expressed in at least four samples, by a detection *p*-value < 0.01). The quality grade of probes was assigned using IlluminaHumanv4.db v1.26.0 package, and additional removal of “bad” probes (probes that match repeat sequences, intergenic or intronic regions, or probes unlikely to provide a specific signal for any transcript) and “no match” probes (probes not matching any genomic region or transcript) was done. The duplicateCorrelation function was used in a random effect approach, to handle blocking on the same subject [28], while the arrayWeights function was applied to down-weight lesser-quality arrays, thus enhancing the accuracy and power of the differential expression analysis [29]. Differentially expressed genes (DEGs) were determined for the defined contrast matrix (comparisons of points before and after the analyzed treatments) based on an analogic design matrix, by using the linear model fit in conjunction with empirical Bayes statistics, which was based on a moderated *t*-test of the *limma* package [25]. Differential expression of genes was defined at an adjusted *p*-value ˂ 0.05 (Benjamini and Hochberg’s, BH, method of adjustment for multiple testing was used) and absolute logarithmic fold change (|log_2_FC|) > 0.5. Expression data were stored within the Gene Expression Omnibus (GEO) repository under accession No GSE145034, using GEO Data Submission Report Plug-in v2.0.0 for GenomeStudio software (Illumina Inc., San Diego, CA, USA).

### 2.7. Bioinformatic Analysis

The computational method Gene Set Enrichment Analysis (GSEA) [30] was applied for detection of statistically significant concordant expression differences in a priori defined sets of genes, between two biological states (herein: before and after treatment). The GSEA includes the whole transcriptome dataset, instead of arbitrarily determined DEGs, allowing the identification of even subtle gene expression changes with biological significance. The subset of a gene set members, which contributes most to the enrichment score (ES), is defined as the leading-edge subset, being also presented as the core of a gene set that accounts for the enrichment signal. The current enrichment analysis of the PBMC transcriptome was employed for an overview of the enriched biological processes. It involved Hallmark gene sets (collection of gene sets obtained by assembling many database gene sets to show well-defined biological processes) exported from the online Molecular Signatures Database (MsigDB) v6.1, with the chip annotation file ilmn_HumanHT_12_V4_0_R1_15002873_B.chip used to collapse each probe set of the expression dataset into a single vector for each gene.

For a more integrative information about treatment effects, the enrichment analysis was performed on the sets of identified DEGs by applying an online functional annotation tool DAVID v6.8 (Database for Annotation, Visualization and Integrated Discovery) [31], using Gene Ontology (GO) and Kyoto Encyclopedia of Genes and Genomes (KEGG) databases. The associated GO terms having an EASE score (modified Fisher’s exact test *p*-value) < 0.1 were considered significantly enriched in DEGs and, hence, were further interpreted in the GO enrichment analysis. 

### 2.8. Validation of PBMC Gene Expression by Reverse Transcription Quantitative Polymerase Chain Reaction (RT-qPCR)

Validation of identified transcriptomic differences (after vs. before each analyzed treatment period) was performed by RT-qPCR, to measure expression of the selected genes. The criteria for selection of genes to be validated were based on the defined cutoff parameters of differential gene expression, as well as on bioinformatic analysis. Validation of expression was done for the following genes: *IL1B*, *TNF*, *IL8*, *RGS1*, *OSM*, and *DUSP2*. The analysis included 45 treatments: 18 AMJ, 18 dAMJ, and nine PLB, in 19 subjects. There were nine subjects with AMJ → dAMJ and eight subjects with dAMJ → AMJ successive treatment transitions (Table S1, Supplementary Materials).

The total RNA (500 ng) extracted from each PBMC sample was treated with DNAseI (Thermo Fisher Scientific Inc., Waltham, MA, USA), to avoid contamination with genomic DNA; subsequently, reverse transcription was done by using a RevertAid First Strand complementary DNA (cDNA) Synthesis kit (Thermo Fisher Scientific Inc., Waltham, MA, USA), with Oligo(dT)18 primer. Real-time qPCR reactions were performed in duplicate on an Applied Biosystems Real-time 7500 system (Applied Biosystems, Foster City, CA, USA), using TaqMan® gene expression assays (Applied Biosystems, Foster City, CA, USA) for the target genes: *IL1B* (Hs01555410_m1), *TNF* (Hs00174128_m1), *IL8* (Hs00174103_m1), *RGS1* (Hs01023772_m1), *OSM* (Hs00171165_m1), and *DUSP2* (Hs00358879_m1), and a reference gene, *PPIA* (Hs99999904_m1). As it is stably expressed, *PPIA* was used to normalize the expression of target genes. The relative expression (mRNA) levels of each target gene were quantified as the mean normalized expression, based on the comparative Ct method [32].

### 2.9. Statistical Analysis

The distribution of continuous variables, including age, BMI, serum glucose, triacylglycerols, total cholesterol, LDL-C, HDL-C, and relative mRNA expression levels of the validated genes, was tested by Shapiro–Wilk’s test. Within-group comparisons (after vs. before each treatment period) were done using *t*-test for dependent samples or Wilcoxon matched pairs test, while between-group comparisons were done by *t*-test or Mann–Whitney U test, for normal or skewed distribution, respectively. In the statistical tests, *p*-values < 0.05 were considered statistically significant. The statistical analysis was accomplished using Statistica 8.0 software package (StatSoft, Inc. 1984-2007).

## 3. Results

### 3.1. Determination of PBMC DEGs for the Applied Treatments

The DEGs identified by analyzing the PBMC transcriptome of study subjects, according to applied treatments, are shown in Table 1 and Table S2 (Supplementary Materials). Two treatments, PLB TP1 (comparison after vs. before TP1) and dAMJ TP2 (after vs. before TP2), resulted in no DEGs. The largest number of DEGs was determined for AMJ TP3 (after vs. before TP3). For single treatments, as well as for successive treatment transitions, most DEGs were upregulated (Table S2, Supplementary Materials).

**Figure 2:**
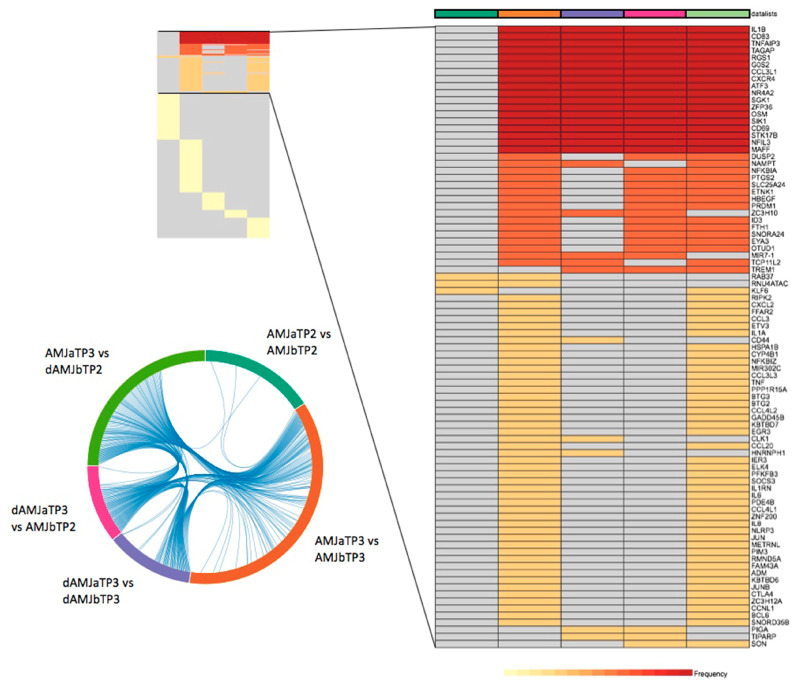
Frequency of overlap of differentially expressed genes in the study subjects, between different treatments/treatment periods. AMJ—treatment with original *Aronia melanocarpa* juice containing a total polyphenol amount of 11,771.09 mg gallic acid equivalent (GAE)/L, which corresponds to 1.17 g of total polyphenols per 100 mL of the allocated treatment (high dose); dAMJ—treatment with aronia juice-based beverage, made by diluting the AMJ with placebo beverage (ratio 1:3) and containing a total polyphenol amount of 2942.77 mg GAE/L, which corresponds to 0.29 g of total polyphenols per 100 mL of the allocated treatment (low dose); aTP—after treatment period; bTP—before treatment period.

According to the heatmap of DEG overlap (Figure 2), the highest number of common DEGs are shared between AMJ TP3 and dAMJ → AMJ successive treatment transition (after TP3 AMJ vs. before TP2 dAMJ). Moreover, there is a noticeable DEG overlap between dAMJ TP3 and AMJ → dAMJ successive treatment transition (after TP3 dAMJ vs. before TP2 AMJ). The overlap is negligible between AMJ TP2 and every other treatment/successive treatment transition (Figure 2).

### 3.2. GSEA Analysis of PBMC Transcriptome

**Figure 3:**
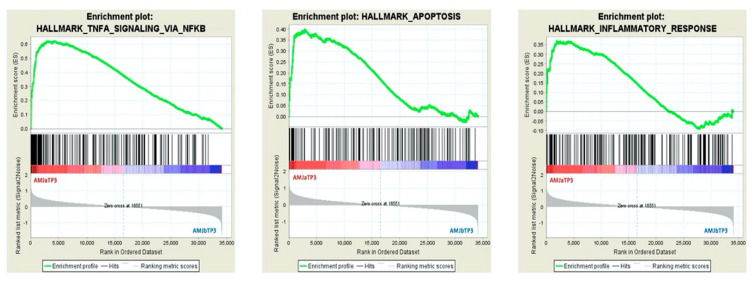
Enrichment plots of top three enriched Hallmark gene sets for the phenotype AMJ aTP3, compared to phenotype AMJ bTP3. Each enrichment plot shows profile of the running enrichment score and positions of gene set constituents on the rank ordered list; AMJ—treatment with original *Aronia melanocarpa* juice containing a total polyphenol amount of 11,771.09 mg gallic acid equivalent (GAE)/L, which corresponds to 1.17 g of total polyphenols per 100 mL of the allocated treatment (high dose); aTP—after treatment period; bTP—before treatment period.

The GSEA results for the whole PBMC transcriptome datasets are given in Table 2 and Figure 3. The analysis disclosed 10 Hallmark gene sets significantly enriched and upregulated after TP3 AMJ, in comparison to before TP3 AMJ (Table 2). Among them, the top three enriched gene sets are TNFA signaling via NFKB, apoptosis, and inflammatory response. The enrichment plots of these three gene sets are shown in Figure 3. TNFA signaling via NFKB was the only Hallmark gene set significantly enriched, after vs. before TP3 dAMJ (Table 2). There were no Hallmark gene sets significantly enriched in AMJ TP2 (Table 2).

### 3.3. Enrichment Analysis of DEGs

The results of enrichment analysis of DEGs are presented in Table S3 (Supplementary Materials). The analysis involved all DEGs and was performed in the categories of GO Biological Process (BP) (GO: BP) and KEGG pathway terms. As expected, based on total number and overlapping of DEGs (Table 1, Figure 2), the most numerous terms, as well as overlapping terms, significantly enriched in DEGs, were found for AMJ TP3 and dAMJ → AMJ successive treatment transition. Among the top-overlapping significantly enriched biological terms were primarily those related to immunity and also those related to regulation of cell proliferation and death, including the following processes: inflammatory response, cellular response to tumor necrosis factor, negative regulation of cell proliferation, immune response, apoptotic process, monocyte chemotaxis, neutrophil chemotaxis, cellular response to interleukin-1, and lymphocyte chemotaxis. The top-overlapping enriched KEGG pathways, TNF signaling pathway, Toll-like receptor signaling pathway, NF-kappa B signaling pathway, cytokine–cytokine receptor interaction, and chemokine signaling pathway, were in line with GSEA analysis performed on the entire transcriptome, thus confirming the true origin of treatment effect from identified DEGs. The latter biological processes and pathways were also depicted amid the top-overlapping in the following comparisons: between AMJ TP3 and dAMJ TP3, between dAMJ TP3 and AMJ → dAMJ successive treatment transition, and between dAMJ → AMJ and AMJ → dAMJ transitions. For each of these three comparisons, most of the significantly enriched biological terms overlapped. The PBMC response specific for AMJ TP3 encompassed several DEG-enriched processes related to metabolism and reactive oxygen species: response to glucocorticoid, sequestering of triglycerides, negative regulation of lipid storage, cellular response to oxidative stress, and regulation of reactive oxygen species metabolic process (Table S3, Supplementary Materials). Only a few general processes (protein phosphorylation, protein autophosphorylation, transcription from RNA polymerase II promoter, and protein K63-linked deubiquitination) overlapped between AMJ TP2 and other treatments/successive treatment transitions. The non-overlapping significantly enriched biological terms found for all stated comparisons were mostly related also to general molecular processes. With regard to the present enrichment analysis, it could be concluded that there are no specific effects of dAMJ → AMJ and AMJ → dAMJ transitions and, therefore, these two successive treatment transitions were excluded from validation of the identified transcriptomic differences.

### 3.4. RT-qPCR Validation of Selected DEGs

Validation of identified PBMC transcriptomic differences, for applied treatments, comprised the RT-qPCR relative expression quantification of the following DEGs: *IL1B*, *TNF*, *IL8*, *DUSP2*, *OSM*, and *RGS1* (Table 3). For all analyzed treatments except AMJ TP2, the selected DEGs had a significantly increased expression, demonstrated through log_2_FC and adjusted *p*-value (Table S2, Supplementary Materials), as well as an involvement in differentially affected biological processes and pathways of interest, related to immunity or regulation of cell proliferation/death and overlapped between the treatments (Table S3, Supplementary Materials). As shown in Table S4 (Supplementary Materials), the vast majority of microarray results were replicated by qPCR, including the comparisons after vs. before: TP2 AMJ, TP2 dAMJ, and TP3 AMJ. The comparison after vs. before TP3 dAMJ showed a significant upregulation of three genes (*TNF*, *IL8*, and *DUSP2*) by qPCR, while microarray resulted in no significant differences regarding the expression of above-mentioned genes. More interestingly, microarray revealed no DEGs for the comparison after vs. before TP1 PLB (placebo), while four of six genes selected for validation (*IL8*, *DUSP2*, *OSM*, and *RGS1*) were significantly upregulated by qPCR. Nevertheless, the significant values of qPCR fold change of expression were evidently lower for placebo, compared to other treatments (Table S4, Supplementary Materials). This means that, if placebo affected the gene expression, these effects were considerably weaker in comparison to the effects of polyphenol-containing aronia juice treatments. Yet, as both low-dose and high-dose polyphenol treatments contained all placebo components, and significant expression changes of the above-mentioned genes were not detected by microarray/qPCR for either of the two polyphenol-containing treatments in TP2, it is unlikely that placebo components had an impact on gene expression. The significant gene expression changes could be due to a lack of the run-in period preceding the TP1.

### 3.5. Effects of the Applied Treatments on Cardio-Metabolic Parameters and Leukocyte Count 

In subjects involved in microarray analysis, comparisons of effects after vs. before each treatment (AMJ, dAMJ, PLB) in each of three treatment periods, with regard to anthropometric and metabolic parameters (BMI, serum glucose, triacylglycerols, total cholesterol, HDL-C, LDL-C) and leukocyte count (total leukocytes, % mononuclear cells, % lymphocytes, % monocytes, % granulocytes), resulted in no significant differences (*p* (*t*-test for dependent samples/Wilcoxon matched pairs test) > 0.05), with exception of HDL-C after vs. before TP2 AMJ (1.4 ± 0.4 mmol/L vs. 1.5 ± 0.4 mmol/L; *p* (*t*-test for dependent samples) = 0.002) (data not shown) and glucose after vs. before TP3 dAMJ (4.5 ± 0.3 mmol/L vs. 4.9 ± 0.2 mmol/L, *p* (*t*-test for dependent samples) = 0.008) (Table S5, Supplementary Materials). Both HDL-C and glucose levels were in the clinical ranges. The comparisons of treatment effects toward cardio-metabolic parameters and leukocyte count are presented for TP3 (Tables S5 and S6, Supplementary Materials), as the period characterized by the pronounced and specific effects of aronia juice consumption on gene expression changes.

## 4. Discussion

In nutrigenomics, gene expression profiling is used to investigate transcriptional changes indicative of mechanisms associated with nutrients and diet. In this study, we demonstrated that the consumption of two different single doses of polyphenol-containing *Aronia melanocarpa* juice during the two consecutive four-week treatment periods had additive effects on PBMC gene expression profiles in subjects at risk of CVD. Comparison of after vs. before the second treatment period (TP2) resulted in the DEG-enriched biological terms depicting an unspecific cellular response, mainly related to general molecular processes, solely for the high-dose polyphenol aronia juice treatment (AMJ), while consumption of low-dose polyphenol juice (dAMJ) resulted in no DEGs. The most pronounced and specific effect of aronia juice consumption on gene expression changes was noticed in the group that received high-dose polyphenol aronia juice in the last treatment period (TP3) of the six-month trial. Likewise, although after acute intake, extra virgin olive oil rich in polyphenols had the strongest impact on modulation of transcription of genes in healthy subjects, while weaker effects were revealed after low-polyphenol extra virgin olive oil consumption [33].

A multitude of overlapping DEGs and biological processes and pathways were observed between high-dose and low-dose treatments in the last treatment period, although the low-dose aronia juice intake had a weaker impact on differential gene expression. The top-overlapping significantly enriched biological processes for high- and low-dose polyphenol aronia juice were related to immunomodulation: immunity and immune response, regulation of cell proliferation, death and apoptosis, inflammatory response, chemotaxis of immune cells, and cellular response to tumor necrosis factor. Pathway analysis, after TP3 high-dose polyphenol intake, confirmed the enrichment in DEGs of the TNF signaling pathway, Toll-like receptor signaling pathway, NF-kappa B signaling pathway, cytokine–cytokine receptor interaction, and chemokine signaling pathway.

Distinct upregulation of *IL1B* and *TNF* was detected exclusively after two consecutive treatment periods. This result indicates a molecular shift toward a proinflammatory environment after the long-term consumption of polyphenol-rich beverage, which is not a favorable scenario with regard to CVD. There is in vitro evidence that treatment of immune cells with resveratrol resulted in either up- or downregulation of expression of TNF, IL1B, and IL8 in a cell- and context-dependent manner [34]. In line with our findings, the in vitro studies revealed that treatments with polyphenols were able to increase the constitutive and/or stimulated expression of these genes [12,13,14,15,16]. In cultured human PBMC, treatment with resveratrol and TLR agonists potentiated the production of TNF, along with NF-κB pathway activation [12], while cocoa procyanidins caused an increase in TNF and IL1B in both resting and phytohemagluttinin-stimulated cells [13,14]. The treatment of normal human epidermal keratinocytes with resveratrol (alone or in combination with TNF) induced a delayed, long-lasting over-expression of IL8 [16], a known proinflammatory marker. The majority of previous studies presented in vitro data with respect to application of polyphenols, yet, the clinical studies utilizing foods or beverages offered a more realistic assessment of the real-life effects. During the last decade, there was a persistent need for studies like ours, which improve knowledge on the molecular background of potential polyphenol effects. In a nine-week interventional trial on obese individuals, IL1B levels were significantly higher in supernatants of LPS-activated PBMC from the individuals who were fed grape compared to those fed placebo, for a three-week period [15]. The stated data indicate that dietary plant products, which contain polyphenols, modulate the inflammatory process in subjects with pre-stimulated inflammatory cells. The observation that resveratrol induced the expression of TNF in TLR agonist-stimulated PBMC indicates that the increased TNF plasma levels detected in healthy individuals 24 h after the treatment with resveratrol could be owing to local immunopotentiation by TLR-stimulating factors from the gut microenvironment, suggesting that consumption of polyphenol-containing products might stimulate leukocyte activity in the gut [12]. Herein, we did not detect any significant change in leukocyte count after TP3. The increased release of IL1B and IL6 from the LPS-stimulated monocytes of the healthy obese individuals after three-week consumption of grape powder was related to the stimulatory effects of these two cytokines on macrophage- and neutrophil-mediated phagocytosis of pathogens [15]. The authors proposed that grape consumption-associated sensitization of the monocytes of obese subjects to raise the production of proinflammatory cytokines in response to a bacterial stimulus might be favorable, as the obese are at an increased risk of infections [15]. Given the established multifactorial nature of a cellular and systemic response to polyphenol treatments, it is crucial to discuss our results in terms of facilitation of applicability of treatments achievable by dietary means. The high dose polyphenols consumed in this study would hardly be achievable by dietary means, but it provides novel data on unexpected proinflammatory gene expression changes, after two consecutive treatment periods. Through the prism of CVD, a previous study suggested that, while the activation of immune responses may stimulate atherosclerosis, the selective activation of immune functions may inhibit arterial inflammation [35]. Use of a low-dose polyphenol-rich aronia juice, which is achievable by food-based dietary choices, also reflected upregulation of particular currently validated genes (*IL1B* and *TNF*), but only one enriched Hallmark gene set. Recognizing that, in advanced atherosclerosis, a shift toward expression of CD4 + Th2 lymphocytes over Th1 (mainly proinflammatory) happens [36], suggesting the adaptation of the immune system to less proinflammatory, is important for the further research on the topic. In alignment is the fact that oxLDL has both a proinflammatory and an immunostimulatory role and that immunization of hypercholesterolemic animals with oxLDL exerts an inhibitory action toward atherosclerosis [37].

In addition to common proinflammatory genes, transcriptome analysis of the last treatment period showed that *OSM*, *RGS1*, and *DUSP2* were among the top DEGs following consumption of aronia juice with a high dose of polyphenols, whereas *OSM* and *RGS1* were also among top DEGs after low-dose polyphenol treatment. Bioinformatic analysis revealed that both *OSM* and *RGS1* participate in inflammation together with *TNF* and *IL1B*. DEG lists generated after both doses of polyphenols showed enrichment in negative regulation of cell proliferation, where *OSM* was a member. OSM is an IL6-type cytokine that has both pro- and anti-inflammatory properties [38]. In diabetic mice, it was shown that treatment with OSM alleviated cardiac ischemia/reperfusion injury through inhibition of cardiomyocyte apoptosis and enhancement of mitochondrial biogenesis and mitochondrial function, while an adverse outcome followed by impairment of insulin sensitivity was demonstrated in OSM receptor knockout diabetic mice [39]. This indicates a protective role of OSM in the cardiometabolic disorders. RGS1 has a regulatory role in the migration of T and B cells and macrophages [40]. Interferon beta treatment upregulated *RGS1* gene expression, which suggests the role of RGS1 in migration of activated immune cells to sites of inflammation, through the regulation of a cytokine–chemokine harmonization mechanism [41]. Upregulation of RGS1 in peripheral blood T lymphocytes decreased their chemotactic response to CXCL12 and CCL19, both implicated in atherosclerosis [40]. As many other molecular components, which are involved in the onset and progression of CVD, RGS1 might have both beneficial and detrimental effects which are mostly dependent on the phase of disease development [40]. Our enrichment analysis of DEGs identified another intriguing molecular regulator, *DUSP2*, as a member of the MAPK signaling pathway, following the high-dose polyphenol aronia juice consumption in the last treatment period. DUSP2 belongs to the DUSP family of phosphatases that negatively regulate the MAPK superfamily in mammalian cells [42]. *DUSP2* transcription was induced in response to oxidative stress and increased susceptibility to apoptosis in cell culture [43]. Its overexpression was also demonstrated in rat cardiomyocytes during ischemia/reperfusion, while salvianolic acid A, well known for its cardiovascular protective properties, reduced DUSP2 expression [44]. Our study failed to provide evidence that consumption of polyphenol-rich aronia juice altered DUSP2 toward its proposed CVD protective expression profile. Upregulation of *DUSP2* in both TP3 treatments in our study is in agreement with a comprehensive human leukocyte transcriptome analysis, in which its induction in activated immune cells was particularly highlighted [45]. However, a recent study demonstrated that DUSP2 negatively modulated the generation of Th17 cells in inflammation and autoimmune response [46].

The PBMC response specific for high-dose polyphenol aronia juice intake during the last treatment period encompassed several DEG-enriched processes related to metabolism and reactive oxygen species: response to glucocorticoid, sequestering of triglycerides, negative regulation of lipid storage, cellular response to oxidative stress, and regulation of reactive oxygen species metabolic process. The latter processes shared most DEGs with the mentioned immune system-related processes, which reflects their interdependence. Intervention studies on the PBMC transcriptome also depicted changes in transcription of genes implicated in glucose and lipid metabolism [33,47] and release of reactive oxygen species [48], in response to supplementation with polyphenol products. Given that we did not detect significant changes in serum glucose and/or lipids, metabolic processes enriched in DEGs might be associated with change(s) in some intermediate metabolic phenotype(s). Thus, TNF and IL1B, as DEGs shared between the enriched metabolic processes, were found to affect the adipocyte expression of fatty acid receptors [49], and changes in plasma omega-6 polyunsaturated fatty acids and palmitic acid levels were associated with intake of the currently applied polyphenol-containing aronia juice treatments in individuals at cardiovascular risk [50]. The accurate role of intermediate metabolic phenotype changes in the prevention/pathogenesis of cardiometabolic diseases should be investigated in further functional studies. 

The present research is the first to apply transcriptomics methods for identification of gene expression changes associated with consumption of polyphenol-containing aronia juice in humans. Due to inter-individual variability in response to polyphenol treatments [5,51], the findings should be interpreted accordingly, as they could be relevant for the determination of specific subpopulations that would benefit best from the use of polyphenol-containing food products, with respect to the prevention of CVD. Although we presented longitudinal transcriptomic data and, hence, the inter-individual effects could be reduced, our results need to be further replicated. Additionally, future intervention studies should include CVD patients, in order to examine the possible contribution of dietary polyphenols in the treatment of CVD.

A recent paper thoroughly reviewed the effects of foods and nutrients in different dietary scenarios according to CVD risk outcomes, presenting the inconsistent results [52]. It points out many obstacles toward quantification of dietary effects on CVD outcomes. Hence, our study limitations have to be addressed. Increase in participant number is always a demand, and participant self-reporting on treatment consumption certainly represents a study limitation. Polyphenol-rich aronia juice is a mix of bioactive polyphenols (mostly phenolic acids and flavonoids), which limits the effect estimation of a sole nutrient, but single-nutrient recommendation is usually less practical in both public health and clinical practices than the food-based approach [53]. Although we cannot exclude the possibility that intake of polyphenols originating from other foods (e.g., coffee, cocoa products, apples, olives/olive oil, tea, and red wine) [54] could contribute to currently detected gene expression profiles, this possibility was minimized by a study design, which included precise instructions to maintain usual habitual diet and a follow-up on this information during the course of the study.

## 5. Conclusions

The present study provides details on the divergent transcriptome changes induced by prolonged polyphenol-rich aronia juice habitual consumption in subjects at risk of CVD. Our data support the proposed cell-specific and context-dependent effects of polyphenols. We also suggest that dietary plant products that contain polyphenols might exert immunomodulatory effects in a complex and time-related context of different biological pathways. Further intervention studies of prolonged consumption of aronia products, comprising larger sample groups and adjusted doses, are needed to validate our findings and to improve understanding of swinging proinflammatory and immune-stimulatory properties of the discussed target genes. Functional studies are required to elucidate the exact molecular mechanisms via which polyphenol-rich aronia products may exert cardiovascular-related effects. 

## Figures and Tables

**Table 1:** The number of differentially expressed genes in peripheral blood mononuclear cells of the study subjects, identified in different treatment periods.

Comparison	Number of DEGs *
PLB aTP1 vs. PLB bTP1	0
AMJ aTP2 vs. AMJ bTP2	78
dAMJ aTP2 vs. dAMJ bTP2	0
AMJ aTP3 vs. AMJ bTP3	172
dAMJ aTP3 vs. dAMJ bTP3	62
AMJ aTP3 vs. dAMJ bTP2	115
dAMJ aTP3 vs. AMJ bTP2	51

DEGs—differentially expressed genes; * differential gene expression was defined at an adjusted *p*-value < 0.05 (after Benjamini and Hochberg’s correction) and absolute logarithmic fold change (|log_2_FC|) > 0.5; AMJ—treatment with original *Aronia melanocarpa* juice containing a total polyphenol amount of 11,771.09 mg gallic acid equivalent (GAE)/L, which corresponds to 1.17 g of total polyphenols per 100 mL of the allocated treatment (high dose); PLB—treatment with placebo beverage, a formulation that has the same appearance, taste, and nutritional composition of the original aronia juice, but without bioactive polyphenols; dAMJ—treatment with aronia juice-based beverage, made by diluting the AMJ with the PLB (ratio 1:3) and containing a total polyphenol amount of 2942.77 mg GAE/L, which corresponds to 0.29 g of total polyphenols per 100 mL of the allocated treatment (low dose); aTP—after treatment period; bTP—before treatment period.

**Table 2:** Gene sets significantly enriched after the given treatment.

Comparison	Enriched HALLMARK GENE SET	Size	ES	NES	FDR q-val
**AMJ aTP3** vs. AMJ bTP3	HALLMARK_TNFA_SIGNALING_VIA_NFKB	199	0.622	3.05	0.000
	HALLMARK_APOPTOSIS	161	0.399	1.8	0.002
	HALLMARK_INFLAMMATORY_RESPONSE	200	0.370	1.82	0.003
	HALLMARK_INTERFERON_GAMMA_RESPONSE	199	0.370	1.80	0.002
	HALLMARK_INTERFERON_ALPHA_RESPONSE	96	0.354	1.54	0.034
	HALLMARK_P53_PATHWAY	196	0.306	1.49	0.047
	HALLMARK_HYPOXIA	197	0.304	1.48	0.045
	HALLMARK_UV_RESPONSE_UP	158	0.314	1.48	0.041
	HALLMARK_IL6_JAK_STAT3_SIGNALING	87	0.342	1.47	0.039
	HALLMARK_KRAS_SIGNALING_UP	200	0.298	1.46	0.037
**dAMJ aTP3** vs. dAMJ bTP3	HALLMARK_TNFA_SIGNALING_VIA_NFKB	199	0.492	2.35	0.000
**AMJ aTP2** vs. AMJ bTP2	None				

Enriched gene sets were calculated for the phenotype after treatment (bold), compared to phenotype before treatment, in the study subjects; size—number of genes included in the gene set; ES—enrichment score, reflects the degree to which a gene set is over-represented in a phenotype after treatment; NES—normalized enrichment score, the enrichment score for the gene set after it is normalized across the analyzed gene sets; FDR q-val—false discovery rate, the estimated probability that the normalized enrichment score represents a false positive finding; AMJ—treatment with original *Aronia melanocarpa* juice containing a total polyphenol amount of 11,771.09 mg gallic acid equivalent (GAE)/L, which corresponds to 1.17 g of total polyphenols per 100 mL of the allocated treatment (high dose); dAMJ—treatment with aronia juice-based beverage, made by diluting the AMJ with placebo beverage (ratio 1:3) and containing a total polyphenol amount of 2942.77 mg GAE/L, which corresponds to 0.29 g of total polyphenols per 100 mL of the allocated treatment (low dose); aTP—after treatment period; bTP—before treatment period.

**Table 3:** The candidate genes’ expression changes in peripheral blood mononuclear cells of the study subjects, during different treatment periods, observed by RT-qPCR.

Gene	AMJaTP2 vs. AMJbTP2		dAMJaTP2 vs. dAMJbTP2		AMJaTP3 vs. AMJbTP3		dAMJaTP3 vs. dAMJbTP3		PLBaTP1 vs. PLBbTP1	
	FC	*p* *	FC	*p* *	FC	*p* *	FC	*p* *	FC	*p* *
***IL1B***	0.79	0.799	1.01	0.948	26.41 †	**0.025**	47.65 †	**0.021**	1.50	0.173
***TNF***	0.70	0.284	0.96	0.594	23.64 †	**0.012**	16.07	**0.021**	1.32	0.139
***IL8***	0.87	0.508	3.06	**0.021**	9.14 †	0.093	30.11	**0.008**	3.50	**0.008**
***DUSP2***	0.93	0.646	1.04	0.767	5.34 †	**0.036**	4.89	**0.015**	1.58	**0.017**
***OSM***	0.96	0.721	1.08	0.515	3.62 †	**0.025**	3.89 †	**0.001**	2.00	**0.011**
***RGS1***	1.16	0.878	1.1	0.953	4.04 †	**0.036**	4.83 †	**0.001**	1.25	**0.028**

FC—fold change; * *t*-test for dependent samples/Wilcoxon matched pairs test; † significantly different expression level in microarray analysis for the same treatment; AMJ—treatment with original *Aronia melanocarpa* juice containing a total polyphenol amount of 11,771.09 mg gallic acid equivalent (GAE)/L, which corresponds to 1.17 g of total polyphenols per 100 mL of the allocated treatment (high dose); PLB—treatment with placebo beverage, a formulation that has the same appearance, taste, and nutritional composition of the original aronia juice, but without bioactive polyphenols; dAMJ—treatment with aronia juice-based beverage, produced by dilution of the AMJ with the PLB (ratio 1:3) and containing a total polyphenol amount of 2942.77 mg GAE/L, which corresponds to 0.29 g of total polyphenols per 100 mL of the allocated treatment (low dose); aTP—after treatment period; bTP—before treatment period.

## Supplementary Materials

The following are available online at https://www.mdpi.com/2072-6643/12/5/1484/s1: Table S1. Samples for microarray and gene expression validation analyses; Table S2. Differentially expressed transcripts in peripheral blood mononuclear cells of the study subjects, identified in different treatment periods; Table S3. Enrichment analysis of differentially expressed genes in the study subjects, identified in different treatment periods; Table S4. Comparison of expression levels of the target genes in peripheral blood mononuclear cells of study subjects: (a) after vs. before TP2 AMJ, (b) after vs. before TP2 dAMJ, (c) after vs. before TP3 AMJ, (d) after vs. before TP3 dAMJ, (e) after vs. before TP1 PLB; Table S5. Comparison of anthropometric and metabolic parameters in the study subjects before and after treatment period 3 (TP3); Table S6. Comparison of leukocyte count in peripheral blood of the study subjects before and after treatment period 3 (TP3).
