# Supplementary material for: The Effects of Aronia melanocarpa Juice Consumption on the mRNA Expression Profile in Peripheral Blood Mononuclear Cells in Subjects at Cardiovascular Risk
Source: Nutrients. 2020 May 20;12(5):1484. doi: 10.3390/nu12051484 (PMC7285191; doi:10.3390/nu12051484)
Supplement: Supplementary file 1 [file nutrients-12-01484-s001.zip › supplementary-772997/Table S1.doc]

| **Subject** | **Gender** | **Age**  **(years)** | **1^st^ 4-week treatment period (TP1)** | **4-week washout** | **2^nd^ 4-week treatment period (TP2)** | **4-week washout** | **3^rd^ 4-week treatment period (TP3)** |
| --- | --- | --- | --- | --- | --- | --- | --- |
| **1** | **female** | **45** | **PLB** |  | **AMJ #** |  | **dAMJ #** |
| **2** | **female** | **49** | **PLB** |  | **AMJ *** |  | **dAMJ** |
| **3** | **male** | **31** | **PLB** |  | **AMJ #** |  | **dAMJ** |
| **4** | **male** | **46** | **PLB #** |  | **AMJ #** |  | **dAMJ** |
| **5** | **male** | **42** | **PLB #** |  | **AMJ #** |  | **dAMJ** |
| **6** | **male** | **33** | **PLB** |  | **AMJ #** |  | **dAMJ #** |
| **7** | **female** | **38** | **PLB** |  | **dAMJ #** |  | **AMJ #** |
| **8** | **male** | **39** | **PLB** |  | **dAMJ #** |  | **AMJ #** |
| **9** | **female** | **35** | **PLB** |  | **dAMJ #** |  | **AMJ #** |
| **10** | **male** | **37** | **PLB** |  | **AMJ #** |  | **dAMJ #** |
| **11** | **female** | **37** | **PLB** |  | **dAMJ #** |  | **AMJ #** |
| **12** | **male** | **40** | **PLB #** |  | **AMJ** |  | **dAMJ** |
| **13** | **female** | **47** | **PLB** |  | **dAMJ #** |  | **AMJ #** |
| **14** | **female** | **31** | **PLB** |  | **AMJ #** |  | **dAMJ #** |
| **15** | **male** | **42** | **PLB #** |  | **dAMJ** |  | **AMJ** |
| **16** | **male** | **32** | **PLB** |  | **dAMJ #** |  | **AMJ** |
| **17** | **male** | **33** | **PLB** |  | **dAMJ #** |  | **AMJ** |
| **18** | **male** | **40** | **PLB** |  | **dAMJ #** |  | **AMJ #** |
| **19** | **male** | **38** | **PLB** |  | **AMJ #** |  | **dAMJ #** |

**Table S1.** Samples for microarray and gene expression validation analyses.

The samples marked with # and * were used for microarray analysis. The mRNA expression profile was analysed in PBMC from: # blood samples collected before and after each marked treatment, * a blood sample collected after the marked treatment. The gene expression validation analysis included all subjects (samples) on AMJ and dAMJ treatments, in both the 2^nd^ and 3^rd^ treatment period, except subject 14 and 16 in the 3^rd^ treatment period (dAMJ and AMJ, respectively), which was caused by sample dropout due to poor RNA quality. The following subjects on PLB treatment: 4, 5, 9, 11, 12, 13, 14, 15, and 16 were also validated, to comply with the number of samples in AMJ treatment groups. After a dropout of 3 samples due to a lack of collected RNA before PLB, we have randomly selected 5 samples, in addition to 4 samples analysed on microarray. In the validation analysis, relative mRNA expression levels of 8 target genes were determined in PBMC from blood samples collected before and after each analysed treatment period.

AMJ – treatment with original *Aronia melanocarpa* juice containing total polyphenol amount of 11771.09 mg gallic acid equivalents (GAE)/L, which corresponds to 1.17 g of total polyphenols per 100 mL of the allocated treatment (high dose); PLB – treatment with placebo beverage, a formulation that has the same appearance, taste and nutritional composition of the original aronia juice, but without bioactive polyphenols; dAMJ – treatment with aronia juice-based beverage, produced by dilution of the AMJ with the PLB (ratio 1:3) and containing total polyphenol amount of 2942.77 mg GAE/L, which corresponds to 0.29 g of total polyphenols per 100 mL of the allocated treatment (low dose).
