# Supplementary material for: The Effects of Aronia melanocarpa Juice Consumption on the mRNA Expression Profile in Peripheral Blood Mononuclear Cells in Subjects at Cardiovascular Risk
Source: Nutrients. 2020 May 20;12(5):1484. doi: 10.3390/nu12051484 (PMC7285191; doi:10.3390/nu12051484)
Supplement: Supplementary file 1 [file nutrients-12-01484-s001.zip › supplementary-772997/Table S4.doc]

**Table S4.** Comparison of expression levels of the target genes in peripheral blood mononuclear cells of study subjects:

a) after vs before TP2 AMJ

| **Gene name** | **log_2_FC by microarray** | **P‡ value** | **FC by**  **RT-qPCR** | **P† value** |
| --- | --- | --- | --- | --- |
| ***IL1B*** |  | ns | 0.79 | 0.799 |
| ***TNF*** |  | ns | 0.70 | 0.284 |
| ***IL8*** |  | ns | 0.87 | 0.508 |
| ***DUSP2*** |  | ns | 0.93 | 0.646 |
| ***OSM*** |  | ns | 0.96 | 0.721 |
| ***RGS1*** |  | ns | 1.16 | 0.878 |

b) after vs before TP2 dAMJ

| **Gene name** | **log_2_FC by microarray** | **P‡ value** | **FC by**  **RT-qPCR** | **P† value** |
| --- | --- | --- | --- | --- |
| ***IL1B*** |  | ns | 1.01 | 0.948 |
| ***TNF*** |  | ns | 0.96 | 0.594 |
| ***IL8*** |  | ns | 3.06 | 0.021 |
| ***DUSP2*** |  | ns | 1.04 | 0.767 |
| ***OSM*** |  | ns | 1.08 | 0.515 |
| ***RGS1*** |  | ns | 1.1 | 0.953 |

c) after vs before TP3 AMJ

| **Gene name** | **log_2_FC by microarray** | **P‡ value** | **FC by**  **RT-qPCR** | **P† value** |
| --- | --- | --- | --- | --- |
| ***IL1B*** | 3.86 | 4.21 x 10^-8^ | 26.41 | 0.025 |
| ***TNF*** | 2.99 | 7.30 x 10^-5^ | 23.64 | 0.012 |
| ***IL8*** | 4.07 | 0.004 | 9.14 | 0.093 |
| ***DUSP2*** | 1.94 | 3.94 x 10^-8^ | 5.34 | 0.036 |
| ***OSM*** | 1.58 | 3.36 x 10^-5^ | 3.62 | 0.025 |
| ***RGS1*** | 2.26 | 1.16 x 10^-7^ | 4.04 | 0.036 |

d) after vs before TP3 dAMJ

| **Gene name** | **log_2_FC by microarray** | **P‡ value** | **FC by**  **RT-qPCR** | **P† value** |
| --- | --- | --- | --- | --- |
| ***IL1B*** | 2.64 | 0.002 | 47.65 | 0.021 |
| ***TNF*** |  | ns | 16.07 | 0.021 |
| ***IL8*** |  | ns | 30.11 | 0.008 |
| ***DUSP2*** |  | ns | 4.89 | 0.015 |
| ***OSM*** | 1.30 | 0.01 | 3.89 | 0.001 |
| ***RGS1*** | 1.87 | 0.0004 | 4.83 | 0.001 |

e) after vs before TP1 PLB

| **Gene name** | **log_2_FC by**  **microarray** | **P‡ value** | **FC by**  **RT-qPCR** | **P† value** |
| --- | --- | --- | --- | --- |
| ***IL1B*** |  | ns | 1.50 | 0.173 |
| ***TNF*** |  | ns | 1.32 | 0.139 |
| ***IL8*** |  | ns | 3.50 | 0.008 |
| ***DUSP2*** |  | ns | 1.58 | 0.017 |
| ***OSM*** |  | ns | 2.00 | 0.011 |
| ***RGS1*** |  | ns | 1.25 | 0.028 |

TP – treatment period; AMJ – treatment with original *Aronia melanocarpa* juice containing total polyphenol amount of 11771.09 mg gallic acid equivalents (GAE)/L, which corresponds to 1.17 g of total polyphenols per 100 mL of the allocated treatment (high dose); PLB – treatment with placebo beverage, a formulation that has the same appearance, taste, and nutritional composition of the original aronia juice, but without bioactive polyphenols; dAMJ – treatment with aronia juice-based beverage, produced by dilution of the AMJ with the PLB (ratio 1:3) and containing total polyphenol amount of 2942.77 mg GAE/L, which corresponds to 0.29 g of total polyphenols per 100 mL of the allocated treatment (low dose); FC – fold change; ‡ moderated t-test with Benjamini & Hochberg adjustment; † t-test for dependent samples/Wilcoxon matched pairs test; ns – nonsignificant; significant difference for all tests at P ˂ 0.05.
