# Supplementary material for: The Effects of Aronia melanocarpa Juice Consumption on the mRNA Expression Profile in Peripheral Blood Mononuclear Cells in Subjects at Cardiovascular Risk
Source: Nutrients. 2020 May 20;12(5):1484. doi: 10.3390/nu12051484 (PMC7285191; doi:10.3390/nu12051484)
Supplement: Supplementary file 1 [file nutrients-12-01484-s001.zip › supplementary-772997/Table S5.doc]

|  | **AMJ †** | **P*** | **dAMJ †** | **P*** | **AMJ ‡** | **P*** | **dAMJ ‡** | **P*** |
| --- | --- | --- | --- | --- | --- | --- | --- | --- |
| **No. of subjects** | 6 |  | 5 |  | 8 |  | 9 |  |
| **Age (years)** | 39.3 ± 4.1 |  | 36.8 ± 5.4 |  | 38.9 ± 4.3 |  | 40.1 ± 6.0 |  |
| **BMI before treatment** | 24.9 (24.6 − 35.0) ^#^ |  | 26.9 ± 4.0 |  | 25.4 (24.6 − 35.0) ^#^ |  | 27.7 ± 2.5 |  |
| **BMI after treatment** | 25.2 (24.2 − 34.7) ^#^ | 0.75 | 26.8 ± 4.1 | 0.55 | 25.4 (24.2 − 34.7) ^#^ | 0.57 | 27.6 ± 2.4 | 0.41 |
| **GLU before treatment** | 4.7 ± 0.4 |  | 4.9 ± 0.2 |  | 4.6 (4.0 − 6.5) ^#^ |  | 5.0 (4.5 − 7.9) ^#^ |  |
| **GLU after treatment** | 4.5 ± 0.3 | 0.11 | 4.5 ± 0.3 | 0.008 | 4.6 (4.1 − 7.3) ^#^ | 0.89 | 4.7 (3.8 − 7.5) ^#^ | 0.05 |
| **TAG before treatment** | 1.0 ± 0.4 |  | 1.4 ± 1.2 |  | 0.9 (0.4 − 5.7) ^#^ |  | 1.5 (0.6 − 7.0) ^#^ |  |
| **TAG after treatment** | 1.0 ± 0.3 | 0.72 | 1.1 ± 0.5 | 0.45 | 1.1 (0.6 − 5.5) ^#^ | 0.33 | 1.8 (0.6 − 5.4) ^#^ | 0.95 |
| **TC before treatment** | 5.2 ± 0.8 |  | 6.0 ± 0.7 |  | 5.4 ± 1.1 |  | 6.3 ± 0.8 |  |
| **TC after treatment** | 5.5 ± 0.3 | 0.48 | 5.9 ± 0.4 | 0.64 | 5.6 ± 1.1 | 0.47 | 6.4 ± 0.9 | 0.71 |
| **HDL-C before treatment** | 1.5 ± 0.5 |  | 1.7 (0.7 − 1.8) ^#^ |  | 1.4 ± 0.4 |  | 1.3 ± 0.4 |  |
| **HDL-C after treatment** | 1.5 ± 0.4 | 0.79 | 1.6 (0.9 − 1.9) ^#^ | 0.89 | 1.4 ± 0.4 | 0.60 | 1.3 ± 0.4 | 0.66 |
| **LDL-C before treatment** | 3.2 ± 0.6 |  | 3.7 ± 0.6 |  | 3.1 ± 0.7 |  | 3.8 ± 0.9 |  |
| **LDL-C after treatment** | 3.3 ± 0.5 | 0.55 | 3.8 ± 0.4 | 0.63 | 3.3 ± 0.6 | 0.47 | 3.8 ± 0.7 | 0.89 |

**Table S5.** Comparison of anthropometric and metabolic parameters in the study subjects before and after the treatment period 3 (TP3).

Continuous variables with a normal distribution are presented as mean ± standard deviation; continuous variables with a non-normal distribution (^#^) are presented as median (minimum – maximum); **†** subjects involved in microarray analysis in TP3; **‡** subjects involved in qPCR validation of gene expression in TP3; P − values related to the within-group difference upon treatment consumption, significant difference at P < 0.05; ***** t-test for dependent samples/Wilcoxon matched pairs test; AMJ – treatment with original *Aronia melanocarpa* juice containing total polyphenol amount of 11771.09 mg gallic acid equivalents (GAE)/L, which corresponds to 1.17 g of total polyphenols per 100 mL of the allocated treatment (high dose); dAMJ – treatment with aronia juice-based beverage, produced by dilution of the AMJ with the placebo (ratio 1:3) and containing total polyphenol amount of 2942.77 mg GAE/L, which corresponds to 0.29 g of total polyphenols per 100 mL of the allocated treatment (low dose); BMI (kg/m^2^) – body mass index; GLU (mmol/L) – glucose; TAG (mmol/L) – triacylglycerols; TC (mmol/L) – total cholesterol; HDL-C (mmol/L) – high-density lipoprotein cholesterol; LDL-C (mmol/L) – low-density lipoprotein cholesterol.
