# Supplementary material for: The Effects of Aronia melanocarpa Juice Consumption on the mRNA Expression Profile in Peripheral Blood Mononuclear Cells in Subjects at Cardiovascular Risk
Source: Nutrients. 2020 May 20;12(5):1484. doi: 10.3390/nu12051484 (PMC7285191; doi:10.3390/nu12051484)
Supplement: Supplementary file 1 [file nutrients-12-01484-s001.zip › supplementary-772997/Table S6.doc]

|  | **AMJ †** | **P*** | **dAMJ †** | **P*** | **AMJ ‡** | **P*** | **dAMJ ‡** | **P*** |
| --- | --- | --- | --- | --- | --- | --- | --- | --- |
| **No. of subjects** | 6 |  | 5 |  | 8 |  | 9 |  |
| **Total leukocytes (x10^9^/L)**  **before treatment** | 6.0 ± 1.5 |  | 5.9 ± 1.6 |  | 6.5 (4.6 − 8.5) ^#^ |  | 6.3 ± 1.2 |  |
| **Total leukocytes (x10^9^/L)**  **after treatment** | 5.8 ± 1.3 | 0.68 | 6.4 ± 0.9 | 0.41 | 6.1 (4.9 − 8.2) ^#^ | 0.62 | 6.4 ± 1.5 | 0.89 |
| **Mononuclear cells (%)**  **before treatment** | 37.3 ± 6.4 |  | 40.5 ± 8.7 |  | 36.0 ± 6.4 |  | 34.7 ± 8.7 |  |
| **Mononuclear cells (%)**  **after treatment** | 38.5 ± 8.7 | 0.49 | 36.7 ± 6.8 | 0.21 | 36.9 ± 8.1 | 0.50 | 34.8 ± 6.9 | 0.97 |
| **Lymphocytes (%) before treatment** | 32.3 ± 5.9 |  | 34.9 ± 7.9 |  | 31.2 ± 5.6 |  | 29.5 ± 7.7 |  |
| **Lymphocytes (%) after treatment** | 33.9 ± 8.2 | 0.38 | 32.0 ± 6.1 | 0.24 | 32.4 ± 7.6 | 0.37 | 30.1 ± 6.1 | 0.85 |
| **Monocytes (%) before treatment** | 4.9 ± 1.2 |  | 5.6 ± 1.5 |  | 4.8 ± 1.4 |  | 5.1 ± 1.4 |  |
| **Monocytes (%) after treatment** | 4.6 ± 0.6 | 0.47 | 4.8 ± 1.0 | 0.17 | 4.5 ± 0.9 | 0.43 | 4.7 ± 1.3 | 0.35 |
| **Granulocytes (%) before treatment** | 62.7 ± 6.4 |  | 59.5 ± 8.7 |  | 64.0 ± 6.4 |  | 65.3 ± 8.7 |  |
| **Granulocytes (%) after treatment** | 61.5 ± 8.7 | 0.49 | 63.3 ± 6.8 | 0.21 | 63.1 ± 8.1 | 0.50 | 65.2 ± 6.9 | 0.97 |

**Table S6.** Comparison of leukocyte count in peripheral blood of the study subjects before and after the treatment period 3 (TP3).

Continuous variables with a normal distribution are presented as mean ± standard deviation; continuous variables with a non-normal distribution (^#^) are presented as median (minimum – maximum); **†** subjects involved in microarray analysis in TP3; **‡** subjects involved in qPCR validation of gene expression in TP3; P − values related to the within-group difference upon treatment consumption, significant difference at P < 0.05; ***** t-test for dependent samples/Wilcoxon matched pairs test; AMJ – treatment with original *Aronia melanocarpa* juice containing total polyphenol amount of 11771.09 mg gallic acid equivalents (GAE)/L, which corresponds to 1.17 g of total polyphenols per 100 mL of the allocated treatment (high dose); dAMJ – treatment with aronia juice-based beverage, produced by dilution of the AMJ with the placebo (ratio 1:3) and containing total polyphenol amount of 2942.77 mg GAE/L, which corresponds to 0.29 g of total polyphenols per 100 mL of the allocated treatment (low dose).
